# Author Correction: Nuclear lipid droplets derive from a lipoprotein precursor and regulate phosphatidylcholine synthesis

**DOI:** 10.1038/s41467-019-09294-8

**Published:** 2019-03-12

**Authors:** Kamil Sołtysik, Yuki Ohsaki, Tsuyako Tatematsu, Jinglei Cheng, Toyoshi Fujimoto

**Affiliations:** Department of Molecular Cell Biology and Anatomy, Nagoya University Graduate School of Medicine, Nagoya, 466-8550 Japan

Correction to: *Nature Communications* 10.1038/s41467-019-08411-x, published online 28 Jan 2019.

The original version of this Article contained errors in the Abstract and Introduction, whereby CCTα was incorrectly defined as an abbreviation of CDP-choline diacylglycerol phosphotransferase α, instead of CTP: phosphocholine cytidylyltransferase α. This has now been corrected in both the PDF and HTML versions of the Article.

