# Supplementary material for: Nuclear lipid droplets derive from a lipoprotein precursor and regulate phosphatidylcholine synthesis
Source: Nat Commun. 2019 Jan 28;10:473. doi: 10.1038/s41467-019-08411-x (PMC6349838; doi:10.1038/s41467-019-08411-x)
Supplement: Supplementary file 1 — Supplementary Information [file 41467_2019_8411_MOESM1_ESM.pdf]

Nuclear lipid droplets derive from a lipoprotein precursor and regulate phosphatidylcholine synthesis

Sołtysik et al

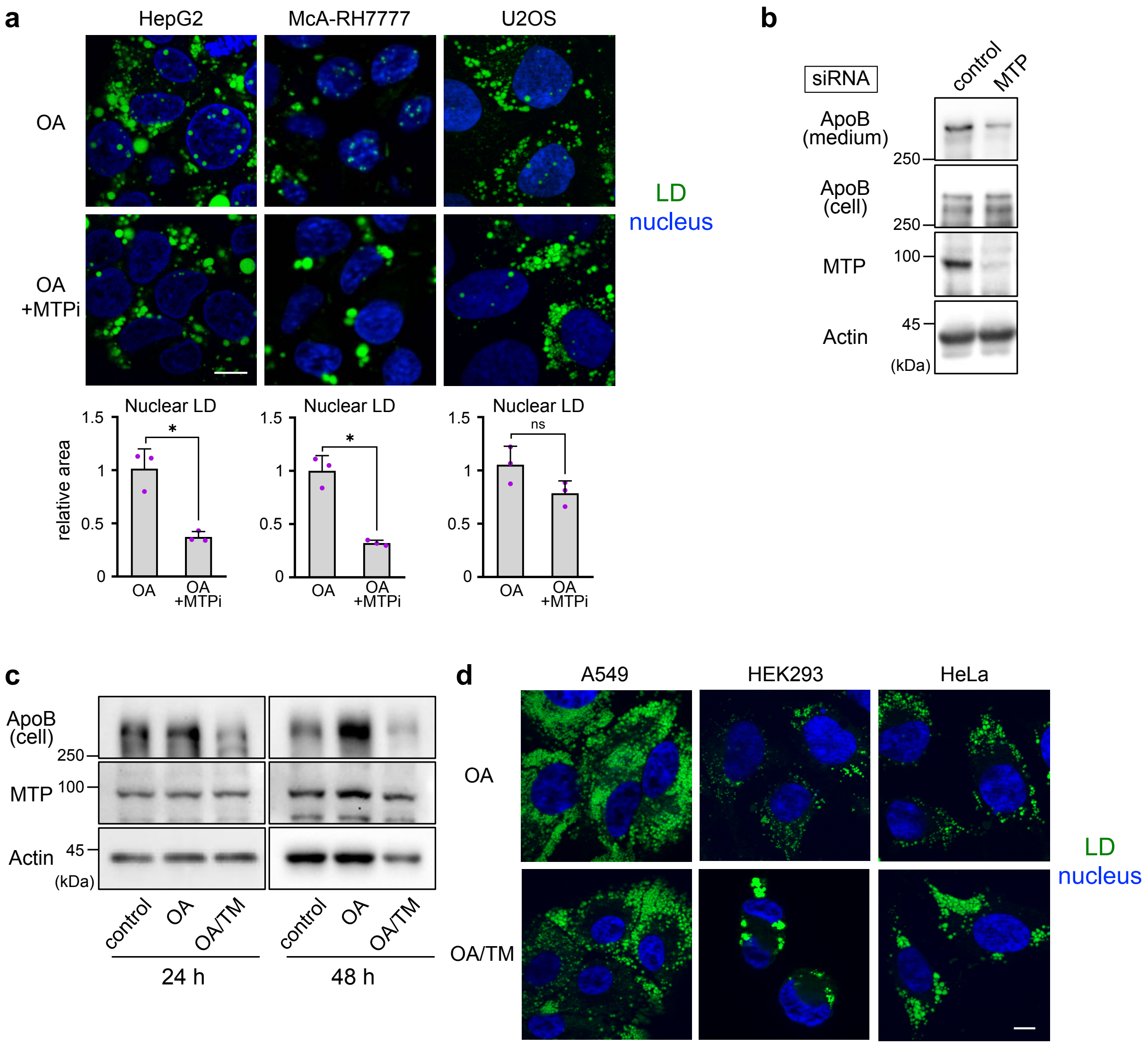

Supplementary Figure 1.

a. MTP inhibition suppressed the increase of nuclear LDs in HepG2, McA-RH7777, but not in U2OS. Cells were treated with 0.4 mM OA in the presence or absence of 1–10 µM BAY 13-9952 for 24 h. LD (green), nucleus (blue). Bar, 10 µm. Mean ± SD of three independent experiments. *$p < 0.01$, Student's t-test.

b. MTP knockdown by RNAi decreased ApoB secretion, but did not affect intracellular ApoB expression in Huh7 cells treated with OA for 24 h. Western blotting.

c. Treatment with 0.4 mM OA and 5 µg/ml tunicamycin (OA/TM) decreased ApoB, but did not affect MTP significantly. Huh7 cells were treated with none, OA, or OA/TM for 24 h or 48 h. Western blotting.

d. Treatment with either OA or OA/TM for 24 h did not increase nuclear LDs in non-hepatocyte cell lines, A549, HEK293, and HeLa. LDs (green), nuclei (blue). Bar, 10 µm.

Source data are provided as a Source data file.

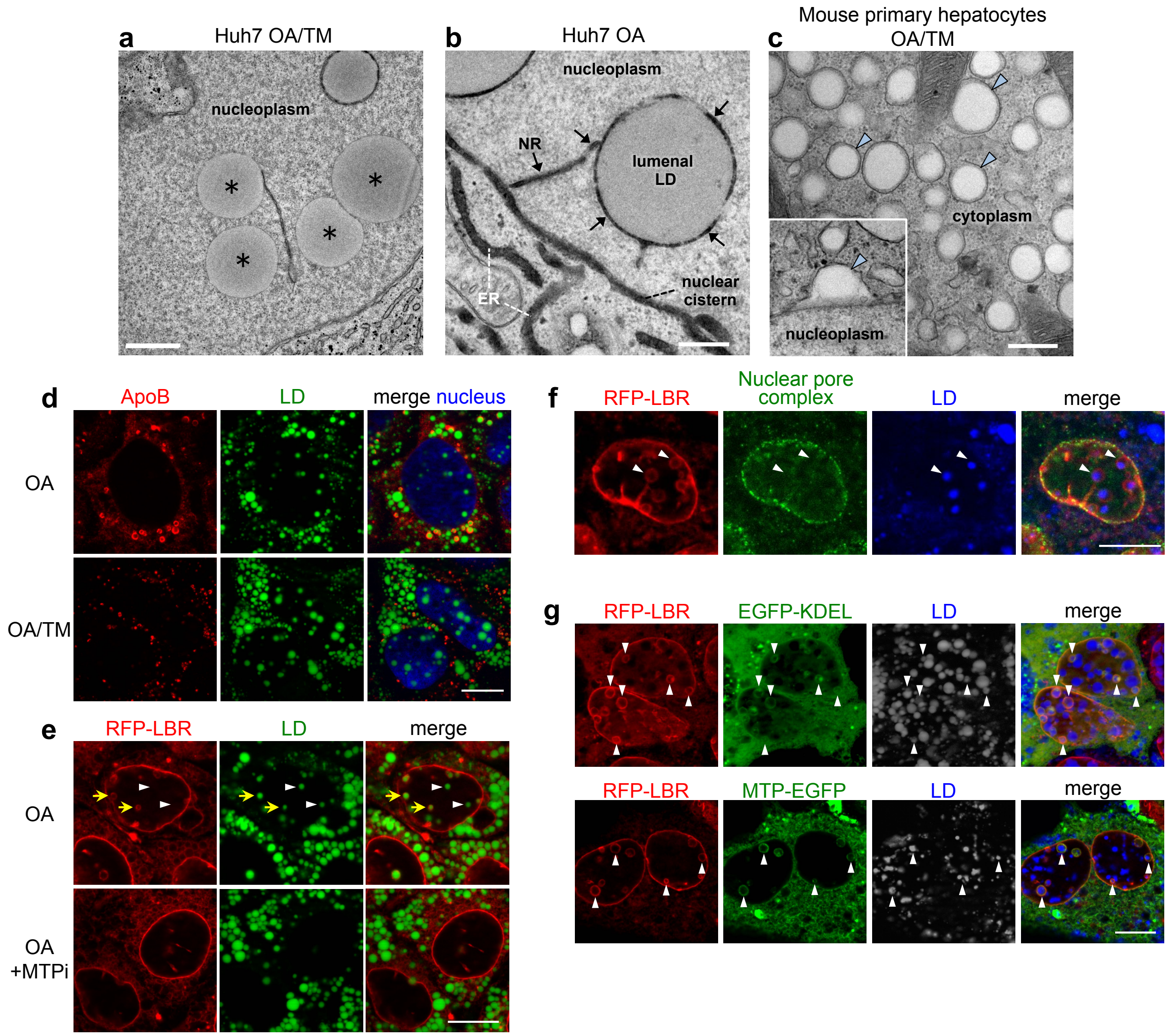

Supplementary Figure 2.

a. Huh7 treated with OA/TM for 24 h harbored many nucleoplasmic LDs (asterisks).

b. Treatment with OA alone generated lumenal LDs in the type I NR lumen (arrows). Huh7 expressing HRP-KDEL was treated with OA for 48 h and incubated with DAB for enzyme histochemistry of HRP.

c. Mouse primary cultured hepatocytes treated with OA and 1 µg/ml TM for 24 h. Lumenal LDs were observed in the lumen of the ER (arrowheads) and the nuclear cistern (arrowhead in inset).

d. Nuclear LDs were not labeled for ApoB. Labeling for ApoB (red) was present in the cytoplasm, but not in the nucleus of Huh7 treated with OA or OA/TM for 48 h. LDs (green), nuclei (blue).

e. Presence of both nucleoplasmic LDs (arrowheads) and NR-lumenal LDs (arrows) in Huh7 treated with OA for 48 h was confirmed by RFP-LBR (red) as the NR marker. Both kinds of LDs were reduced by MTPi (100 nM BAY 13-9952). LDs (green).

f. The nuclear pore complex (green) was scarcely labeled in the nuclear region despite the presence of many RFP-LBR rings (red, arrowheads) in Huh7 treated with OA/TM for 48 h. LDs (blue). This result indicates that most LBR rings are derived from the type I NR.

g. EGFP-KDEL and MTP-EGFP (green), which are soluble proteins in the ER and nuclear cisternal lumen, exhibited ring-shaped colocalization with RFP-LBR (red) around NR-lumenal LDs (arrowheads) in Huh7 treated with OA/TM for 48 h. LDs (blue).

Bars, 0.5 µm (**a–c**); 10 µm (**d–g**).

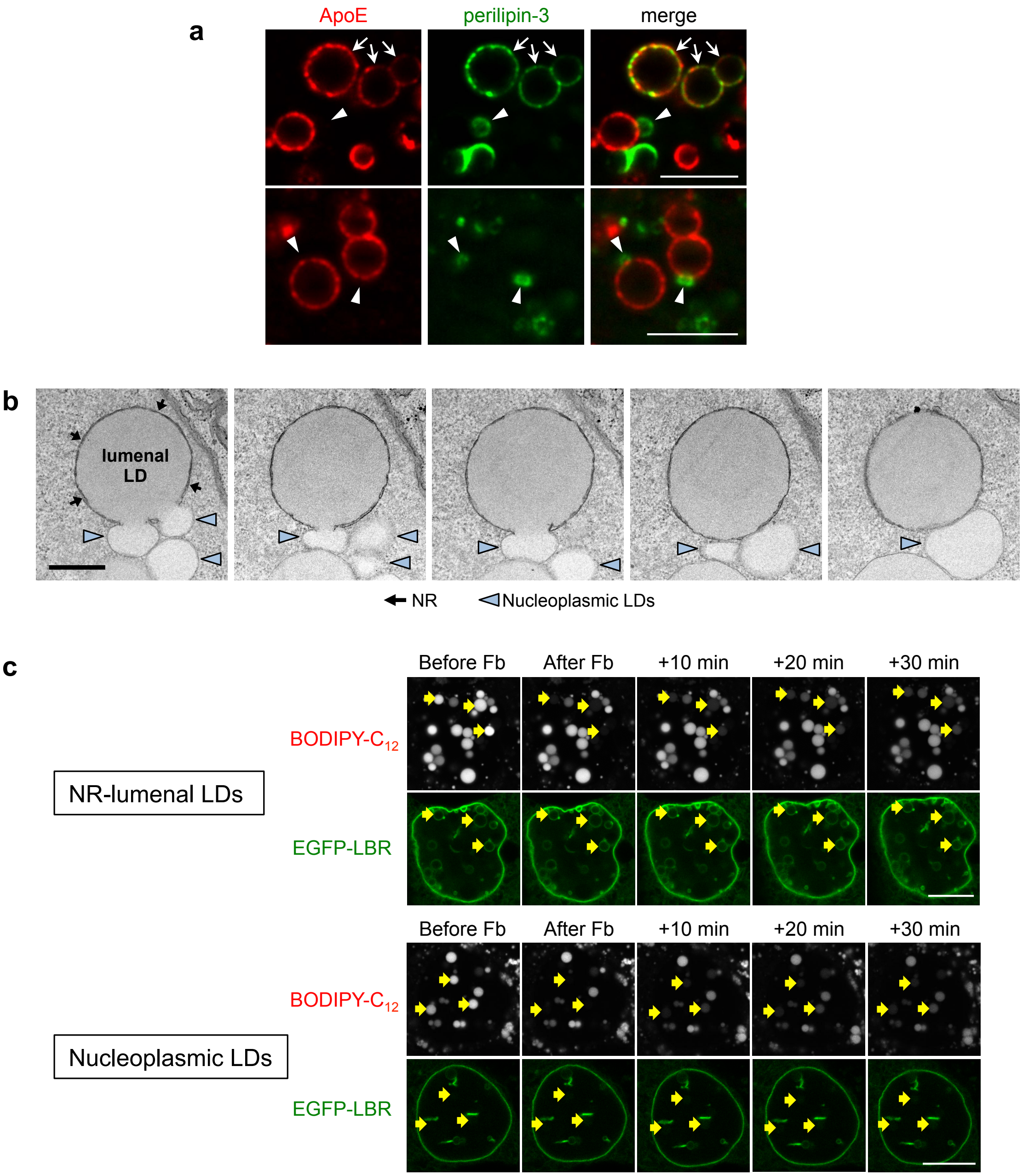

Supplementary Figure 3.

a. Double labeling of ApoE (red) and perilipin-3 (green) in Huh7 treated with OA/TM for 48 h. Perilipin-3 showed colocalization with ApoE around the same LD (arrows) or distributed in small nucleoplasmic LDs (arrowheads) binding to ApoE-positive NR-lumenal LDs. Bars, 5 μm.

b. Serial ultrathin sections to show the coalescence of an NR-lumenal LD with several nucleoplasmic LDs in Huh7 treated with OA/TM for 24 h. Arrows indicate the NR membrane. The LD fusion occurs through defects in the NR membrane. Bar, 0.5 μm.

c. Examples of FRAP sequence. Huh7 was cultured with OA/TM for 48 h and incubated for 1 h with BODIPY558/568-C_{12} to label LDs metabolically. NR-lumenal LDs and nucleoplasmic LDs distinguished by EGFP-LBR rings were photobleached (arrows). For nucleoplasmic LDs, only those that were clearly separated from LBR rings were used for photobleaching. Bars, 10 μm.

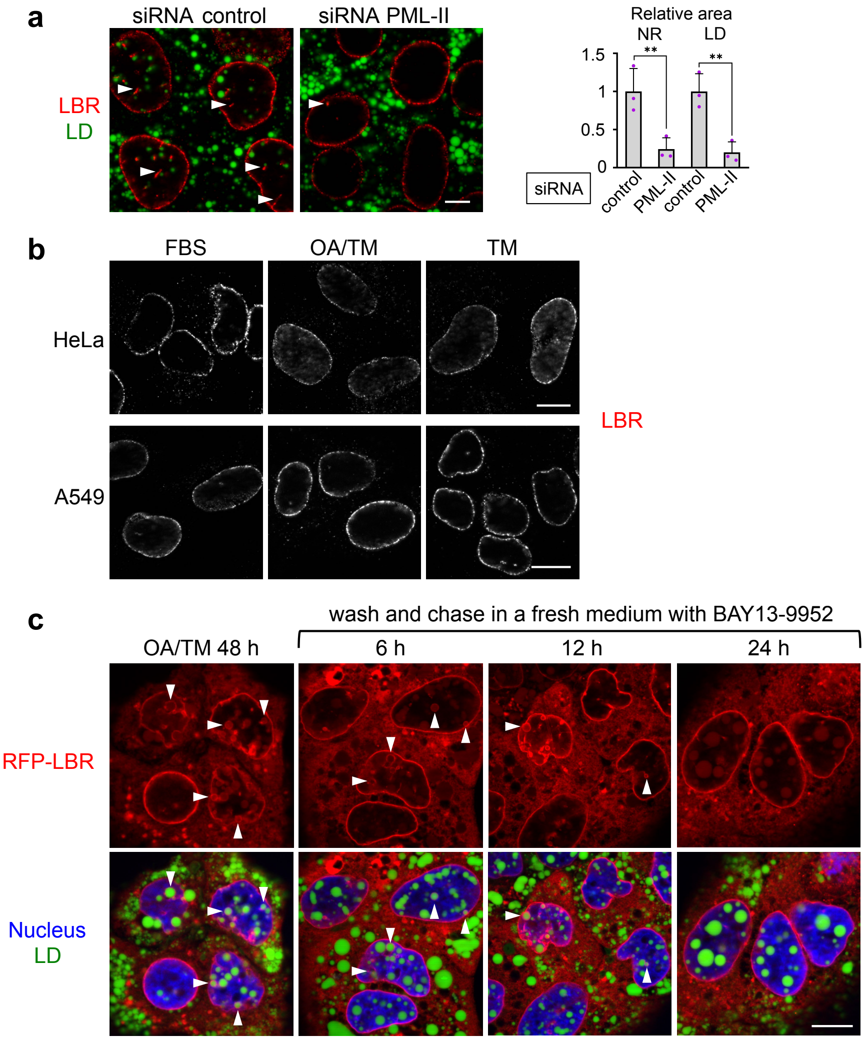

Supplementary Figure 4.

a. PML-II knockdown suppressed the increase of the NR (red) and nuclear LDs (green) in Huh7 treated with OA/TM for 24 h. Endogenous LBR was immunolabeled to observe the NR. Mean ± SD of three independent experiments. $^{**}p < 0.05$, Student's *t*-test.

b. The NR did not increase in HeLa and A549 treated with OA/TM or TM alone for 24 h. Endogenous LBR was labeled.

c. The increase of nucleoplasmic LDs after OA/TM washout was not suppressed by MTP inhibition. Huh7 cells treated with OA/TM for 48 h were chased in a fresh medium without OA/TM, but containing 10 μM BAY 13-9952. Disintegration of the NR membrane (red, RFP-LBR; arrowheads) and the increase of nucleoplasmic LDs (green) occurred even in the presence of MTPi. Nuclei (blue).

Bars, 10 μm. Source data are provided as a Source data file.

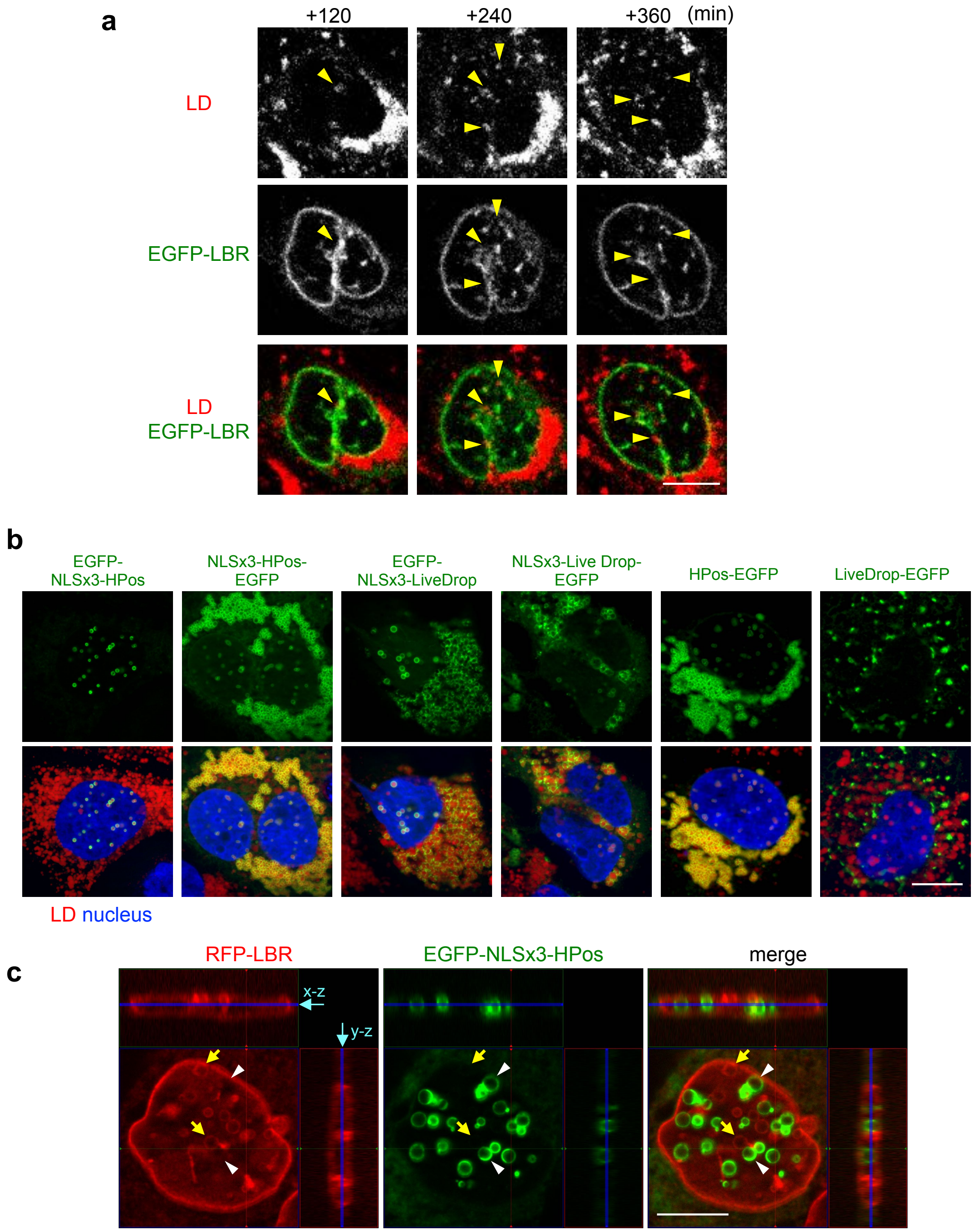

Supplementary Figure 5.

a. Nuclear LDs (red, LipidTox Red) that were regenerated in daughter cells at early time points after mitosis overlapped with the NR (green, EGFP-LBR), indicating that they are NR-lumenal LDs. 0 min = the time point of nuclear envelope reformation. Selected frames from Movie S3.

b. Distribution of various LD marker proteins in Huh7 treated with OA for 24 h. LDs (red, LipidTox Red), nuclei (blue). EGFP-NLSx3-HPos was targeted to nuclear LDs most selectively.

c. EGFP-NLSx3-HPos (green, arrowheads) and RFP-LBR (red, arrows) were segregated from each other in Huh7 treated with OA for 24 h, verifying that EGFP-NLSx3-HPos marks only nucleoplasmic LDs.

Bars, 10 μm.

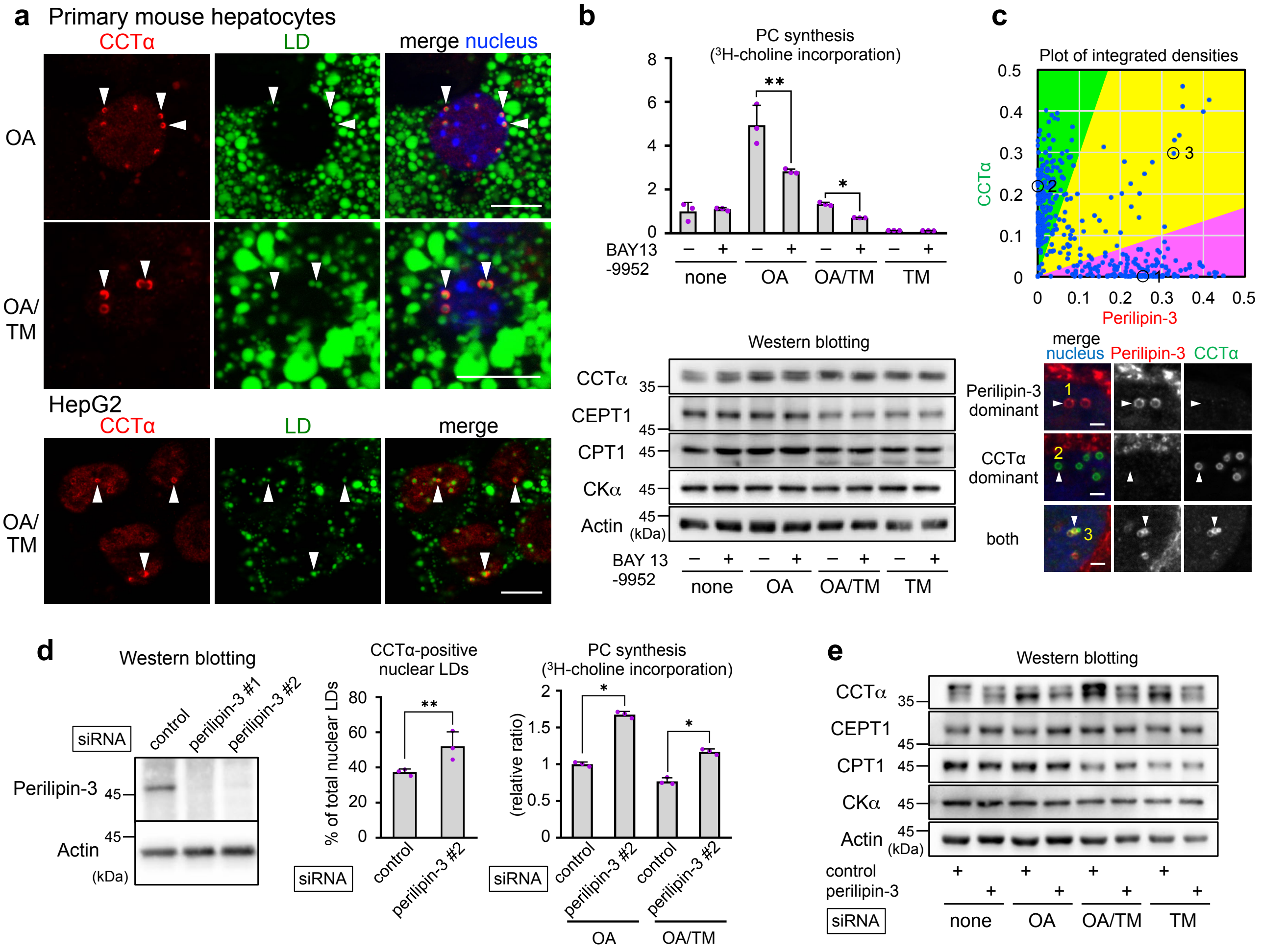

Supplementary Figure 6.

a. Recruitment of endogenous CCTα (red) to nuclear LDs (green) was observed in primary cultured mouse hepatocytes treated with OA or OA/TM for 48 h, and also in HepG2 treated with OA/TM for 24 h (arrowheads). Bars, 10 μm.

b. MTP inhibition decreased PC synthesis significantly, but did not change the expression of CCTα or any other enzymes of the PC synthetic Kennedy pathway. Huh7 treated with none, TM alone, OA alone, or OA/TM for 24 h, with or without 100 nM BAY 13-9952. PC synthesis was measured by ^{3}H-choline incorporation. Mean ± SD of triplicate samples. *$p < 0.01$, **$p < 0.05$, one-way ANOVA followed by Tukey test. Western blotting of CCTα, choline kinase α (CKα), choline/ethanolamine phosphotransferase 1 (CEPT1), and diacylglycerol cholinetransferase 1 (CPT1) is shown. The expression of choline kinase β was negligible.

c. The relative labeling intensity of perilipin-3 and CCTα in individual nucleoplasmic LDs in Huh7 cells treated with OA for 24 h is shown as scatter plot. Nucleoplasmic LDs (n = 396) were classified into three groups: perilipin-3-dominant (the relative intensity of perilipin-3/CCTα > 3), CCTα-dominant (the relative intensity of perilipin-3/CCTα < 1/3), and the other. The proportion of the three groups is shown in the Venn diagram in Fig. 6a. Examples of nucleoplasmic LDs belonging to the three groups are shown. The LDs numbered in the figure (arrowheads) correspond to the dot of the same number in the scatter plot. Bars, 2 μm.

d. Perilipin-3 knockdown. Western blotting confirmed the RNAi effect on expression of perilipin-3. Perilipin-3 knockdown with siRNA #2 increased nuclear LDs harboring CCTα and PC synthesis measured by ^{3}H-choline incorporation. Huh7 was treated with OA for 24 h after siRNA transfection. Mean ± SD of three independent experiments. *$p < 0.01$, **$p < 0.05$, Student's t-test.

e. Perilipin-3 knockdown caused a slight decrease of CCTα, but did not significantly affect CKα, CEPT1, or CPT1. Huh7 was transfected with either control or perilipin-3 siRNA, and treated with none, TM alone, OA alone, or OA/TM for 24 h.

Source data are provided as a Source data file.

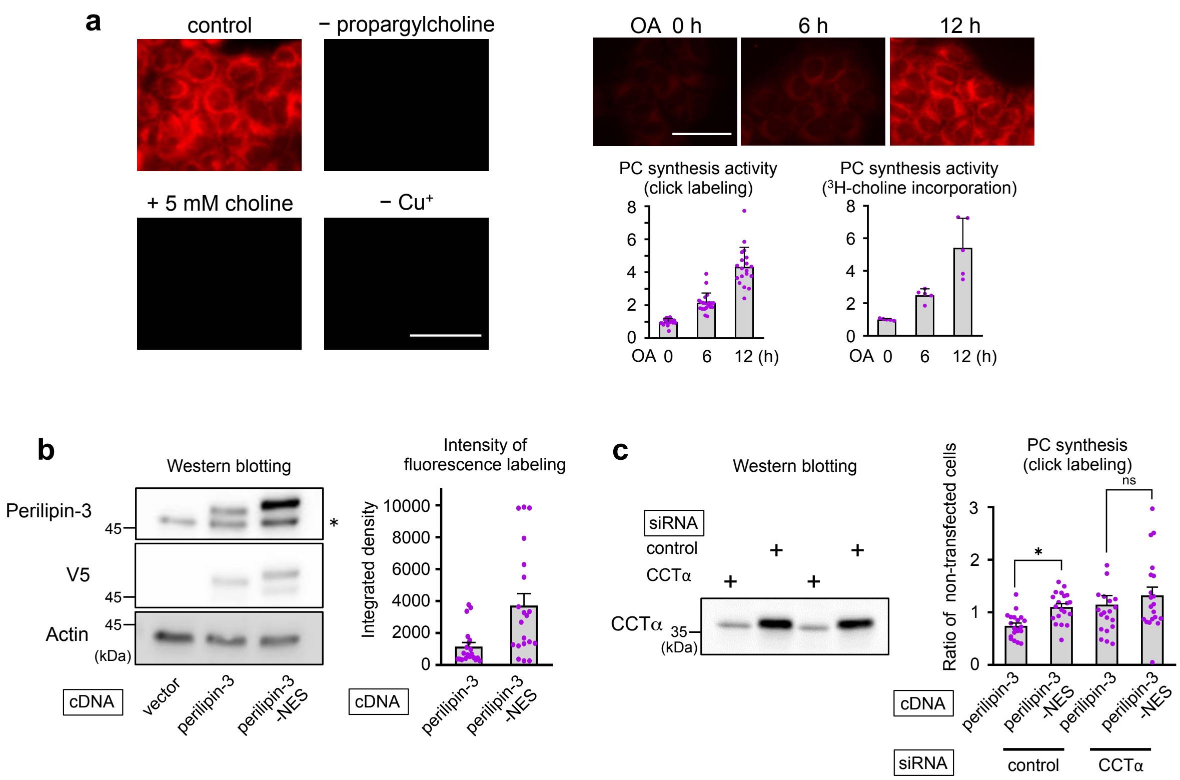

Supplementary Figure 7.

a. Fluorescence imaging of *de novo* PC synthesis by click chemistry. Bars, 50 μm. (Left panel) Experiments to show the specificity of the labeling. Huh7 cells incubated with 0.25 mM propargylcholine exhibited intense fluorescence signal, whereas cells incubated without propargylcholine or those incubated with propargylcholine together with an excess choline (5 mM) were not labeled. Even when cells were incubated with propargylcholine, labeling did not occur by omission of Cu^+ from the click reaction mixture. (Right panel) Cells were cultured with OA for various durations, treated with 0.25 mM propargylcholine for the last 1 h, fixed, and reacted to conjugate Cy3-azide to the alkyne residue by click reaction. This method labels choline-containing phospholipids, which are predominantly PC with this short incubation time[22]. The integrated fluorescence intensity obtained by click PC labeling and PC synthesis measured by ^{3}H-choline incorporation increased with a similar time course in Huh7 cells treated with OA. Mean ± SEM, n = 19 (0 h), 21 (6 h), 19 (12 h) (left); Mean ± SD of quintuplicate samples (right).

b. Both western blotting and the integrated intensity of fluorescence labeling showed that the expression level of perilipin-3-NES was significantly higher than that of the wild-type perilipin-3. *: endogenous perilipin-3. Mean ± SEM of one representative experiment, n = 20.

c. Knockdown of CCTα abolished the difference in PC synthesis caused by the expression of the perilipin-3 constructs. The effect of CCTα knockdown was confirmed by Western blotting. The integrated fluorescence intensity of the click PC labeling was measured as shown in Fig. 6e and presented as the relative ratio to that of non-transfected cells in the same micrograph. Mean ± SEM of one representative experiment, n = 20.

Source data are provided as a Source data file.

Supplementary Table 1. Dilution of Antibodies used for immunofluorescence labeling and Western blotting

Antibody	Source	Catalog number	Dilution for immuno-fluorescence labeling	Dilution for Western blotting
Actin	Sigma	A2066	–	1/5,000
ApoB	Rockland	600-101-111	1/500	1/5,000
ApoC-III	Thermo Fisher	6H21L11	1/100	–
ApoE	Innogenetics	M-012-0500	1/100	–
CCTα	Dr. Neale Ridgway	–	1/2,000	1/2,000
CEPT1	Abgent	#AP10372a	–	1/1,000
CKα	Proteintech	13520-1-AP	–	1/5,000
CKβ	Santa Cruz	sc-398957	–	1/1,000
CPT1	Santa Cruz	sc-515577	–	1/1,000
FLAG	Sigma	F1804	1/1,000	–
Lamin A/C	Cell Signaling	#4777	1/200	–
Lamin B1	Abcam	ab16048	1/1,000	–
Lamin B receptor	Genway Biotech	GWB-C7CA28	1/1,000	–
MTP	Santa Cruz	sc-33116	–	1/1,000
Nucleopore complex	Covance	MMS-120P	1/100	–
Perilipin-3 (Rabbit)	Ohsaki et al (2006)[49]	–	1/1,000	1/5,000
Perilipin-3 (GP)	Progen	GP30	1/200	–
V5	Thermo Fisher	R960-25	1/1,000	1/5,000
Cy3-conjugated donkey anti-mouse IgG	Jackson ImmunoResearch	715-165-151	1/3,000	–
Cy3-conjugated donkey anti-rabbit IgG	Jackson ImmunoResearch	711-165-152	1/3,000	–
Cy3-conjugated donkey anti-guinea pig IgG	Jackson ImmunoResearch	706-165-148	1/3,000	–
Alexa Fluor 488-conjugated donkey anti-mouse IgG	Jackson ImmunoResearch	715-545-150	1/500	–
Alexa Fluor 488-conjugated donkey anti-rabbit IgG	Jackson ImmunoResearch	711-545-152	1/500	–
Alexa Fluor 647-conjugated donkey anti-rabbit IgG	Jackson ImmunoResearch	711-605-152	1/500	
HRP-conjugated goat anti-mouse IgG	Thermo Fisher	31432	–	1/50,000
HRP-conjugated goat anti-rabbit IgG	Thermo Fisher	31458	–	1/50,000
HRP-conjugated donkey anti-goat IgG	Bethyl Laboratories	A50-200P	–	1/10,000

Supplementary Table 2. List of primers and oligonucleotides used in this study

Name	Sequence (5’ – 3’)
MTP-F	CGGTCGACAATATGATTCTTCTTGCTGT
MTP-R	CTGGATCCAAACCATCCGCTGGAAGTACT
HPos-F	AATTCGGTGGTGGTGGTGGTATGGACGTCCTGGTCCCACTCCTGCAGCTGCTGGTGCTGCTTCTCACCCTGCCCCTGCACCTCCTGGCTCTGGGCTGCTGGCAGCCACTCTTTGAAGCGATTGGCAAGATATTCAGCAATATCCGCATCAGCACGCAGAAAGAGATAGGTGGTGGTGGTGGTG
HPos-R	TCGACACCACCACCACCACCTATCTCTTTCTGCGTGCTGATGCGGATATTGCTGAATATCTTGCCAATCGCTTCAAAGAGTGGCTGCCAGCAGCCCAGCAGAGCCAGGAGGTGCAGGGGCAGGGTGAGAAGCAGCACCAGCAGCTGCAGGAGTGGGACCAGGACGTCCATACCACCACCACCACCG
LiveDrop-F	AATTCGGTGGTGGTCACTACGAGTTCATTTCCTGGAAAATCACCTCCATCTGGGTGTTCGGCTTCTTCATCCGCTACGTCATCCTGATGCCCCTCCGGGTATTGGTATGCTTCGTTGGTGTAGTGTGGTTAACAGTCTGCACGGCTGCAGTGGGATACTTGAAGGATGGGCCCTTCAAGCGGGATGGTGGTGGTG
LiveDrop-R	TCGACACCACCACCATCCCGCTTGAAGGGCCCATCCTTCAAGTATCCCACTGCAGCCGTGCAGACTGTTAACCACACTACACCAACGAAGCATACCAATACCCGGAGGGGCATCAGGATGACGTAGCGGATGAAGAAGCCGAACACCCAGATGGAGGTGATTTTCCAGGAAATGAACTCGTAGTGACCACCACCG
NLS-F	TCGAGAAAGAGGCCTGCGGCTACCAAAAAAGCAGGCCAGGCAAAGAAGAAGAAAAAGAGGCCTGCGGCTACCAAAAAAGCAGGCCAGGCAAAGAAGAAGAAAAAGAGGCCTGCGGCTACCAAAAAAGCAGGCCAGGCAAAGAAGAAGAAAG
NLS-R	AATTCTTTCTTCTTCTTTGCCTGGCCTGCTTTTTTGGTAGCCGCAGGCCTCTTTTTCTTCTTCTTTGCCTGGCCTGCTTTTTTGGTAGCCGCAGGCCTCTTTTTCTTCTTCTTTGCCTGGCCTGCTTTTTTGGTAGCCGCAGGCCTCTTTC
NES-F	CCTGCAGCTGCCTCCCCTGGAGCGCCTGACCCTGGACTAAG
NES-R	GATCCTTAGTCCAGGGTCAGGCGCTCCAGGGGAGGCAGCTGCAGGGTAC
