# Supplementary material for: Nuclear lipid droplets derive from a lipoprotein precursor and regulate phosphatidylcholine synthesis
Source: Nat Commun. 2019 Jan 28;10:473. doi: 10.1038/s41467-019-08411-x (PMC6349838; doi:10.1038/s41467-019-08411-x)
Supplement: Supplementary file 9 — Reporting Summary [file 41467_2019_8411_MOESM9_ESM.pdf]

nature research

Corresponding author(s): Toyoshi Fujimoto, Yuki Ohsaki

Reporting Summary

Nature Research wishes to improve the reproducibility of the work that we publish. This form provides structure for consistency and transparency in reporting. For further information on Nature Research policies, see Authors & Referees and the Editorial Policy Checklist.

Statistical parameters

When statistical analyses are reported, confirm that the following items are present in the relevant location (e.g. figure legend, table legend, main text, or Methods section).

n/a	Confirmed	
☐	☒	The exact sample size (n) for each experimental group/condition, given as a discrete number and unit of measurement
☐	☒	An indication of whether measurements were taken from distinct samples or whether the same sample was measured repeatedly
☐	☒	The statistical test(s) used AND whether they are one- or two-sided<br>*Only common tests should be described solely by name; describe more complex techniques in the Methods section.*
☒	☐	A description of all covariates tested
☐	☒	A description of any assumptions or corrections, such as tests of normality and adjustment for multiple comparisons
☐	☒	A full description of the statistics including central tendency (e.g. means) or other basic estimates (e.g. regression coefficient) AND variation (e.g. standard deviation) or associated estimates of uncertainty (e.g. confidence intervals)
☒	☐	For null hypothesis testing, the test statistic (e.g. F, t, r) with confidence intervals, effect sizes, degrees of freedom and P value noted<br>*Give P values as exact values whenever suitable.*
☒	☐	For Bayesian analysis, information on the choice of priors and Markov chain Monte Carlo settings
☒	☐	For hierarchical and complex designs, identification of the appropriate level for tests and full reporting of outcomes
☒	☐	Estimates of effect sizes (e.g. Cohen's d, Pearson's r), indicating how they were calculated
☐	☒	Clearly defined error bars<br>*State explicitly what error bars represent (e.g. SD, SE, CI)*

Our web collection on statistics for biologists may be useful.

Software and code

Policy information about availability of computer code

Data collection	Fusion-Capt Advance Software 16.15,
Data analysis	Microsoft Excel 16.14.1, PQStat 1.4.8, ImageJ 1.52e, Adobe Photoshop CS3, Zeiss Zen 2 (blue edition), Prism 8

For manuscripts utilizing custom algorithms or software that are central to the research but not yet described in published literature, software must be made available to editors/reviewers upon request. We strongly encourage code deposition in a community repository (e.g. GitHub). See the Nature Research guidelines for submitting code & software for further information.

Data

Policy information about availability of data

All manuscripts must include a data availability statement. This statement should provide the following information, where applicable:
- Accession codes, unique identifiers, or web links for publicly available datasets
- A list of figures that have associated raw data
- A description of any restrictions on data availability

Biochemical data and imaging data used for all figures are available upon request.

Field-specific reporting

Please select the best fit for your research. If you are not sure, read the appropriate sections before making your selection.

☒ Life sciences ☐ Behavioural & social sciences ☐ Ecological, evolutionary & environmental sciences

For a reference copy of the document with all sections, see nature.com/authors/policies/ReportingSummary-flat.pdf

Life sciences study design

All studies must disclose on these points even when the disclosure is negative.

Sample size	The sample sizes were determined empirically based on other studies with similar methodologies. No statistical method was used to predetermine the sample size. The sample sizes and statistical methods are described in figure legends and in the method section. Briefly, for biochemical data and quantifications, at least 3 biological replicates were used; for imaging data, at least 3 biological replicates (more than 100 cells in each group) were used unless stated otherwise in legends.
Data exclusions	With common sense, if positive controls were found negative or if negative controls were found positive, data were not analyzed.
Replication	Experiments were repeated at least 3 times and considered as sound when results were similar.
Randomization	Pictures for quantification were taken in random fields. No statistical method for sample selection from the population was used.
Blinding	No blinding was used based on previous studies with similar methodologies.

Reporting for specific materials, systems and methods

Materials & experimental systems

n/a	Involved in the study	
☐	☒	Unique biological materials
☐	☒	Antibodies
☐	☒	Eukaryotic cell lines
☒	☐	Palaeontology
☐	☒	Animals and other organisms
☒	☐	Human research participants

Methods

n/a	Involved in the study	
☒	☐	ChIP-seq
☒	☐	Flow cytometry
☒	☐	MRI-based neuroimaging

Unique biological materials

Policy information about availability of materials

Obtaining unique materials	All unique materials are available from authors.

Antibodies

Antibodies used	Rabbit anti-human perilipin-3 antibody was raised against a peptide of human perilipin-3 segment (amino acids 305–318). Rabbit anti-CCTα antibody was donated by Dr. Neale Ridgway (Dalhousie University). Rabbit anti-lamin B receptor (Genway Biotech GWB-C7CA28), rabbit anti-lamin B1 (Abcam ab16048), mouse anti-lamin A/C (Cell Signaling #4777), rabbit anti-actin (Sigma A2066), and mouse anti-FLAG (Sigma F1804), goat anti-MTP (Santa Cruz sc-33116), mouse anti-ApoE (Immunogenetics M-012-0500), mouse anti-choline kinase ??(CK?) (Santa Cruz sc-398957), mouse anti-diacylglycerol cholinetransferase 1 (CPT1) (Santa Cruz sc-515577), rabbit anti-choline/ethanolamine phosphotransferase 1 (CEPT1) (Abgent #AP10372a), goat anti-ApoB (Rockland 600-101-111), rabbit anti-ApoC-III (Thermo Fisher 6H21L11), mouse anti-V5 (Thermo Fisher R960-25), mouse anti-choline kinase ??(CK?) (Proteintech 13520-1-AP), guinea pig anti-perilipin-3 (Progen GP30), and mouse anti-nucleopore complex (Covance MMS-120P) antibodies were obtained from the respective suppliers. Secondary antibodies conjugated to fluorochromes and peroxidase were purchased from Thermo Fisher, Jackson ImmunoResearch Lab, and Bethyl Laboratories. See Supplementary Table 1 for dilutions of antibodies used for immunofluorescence labeling and Western blotting.
Validation	TAntibodies to human perilipin-3 and V5 (Ohsaki Y, et al., Biochem Biophys Res Commun 347, 279-287, 2006), CCTα (Gehrig K et al., Mol Biol Cell 19, 237-247, 2008), LaminA/C, lamin B receptor, and Flag (Ohsaki Y et al., J Cell Biol 212, 29-38, 2016), ApoB,

MTP, and actin (Ohsaki Y et al., J Cell Sci 121, 2415-2422, 2008) were validated in respective studies. Other commercial antibodies were validated by Western blotting and immunofluorescence microscopy in our lab.

Eukaryotic cell lines

Policy information about cell lines

Cell line source(s)	HepG2 (JCRB1054), HEK293 (JCRB9068), and HeLa (JCRB9004) were obtained from the Japanese Collection of Research Bioresources Cell Bank. McA-RH7777 (CRL-1601) was obtained from ATCC. A549 (Dr. Takashi Takahashi, Nagoya University), Huh7 (Dr. Eija Jokitalo, University of Helsinki) and U2OS (Dr. Hidemasa Goto, Aichi Cancer Center) were provided by the indicated researcher, respectively. Primary cultured mouse hepatocytes were isolated in our labolatory.
Authentication	No particular examination except for morphological observation was done.
Mycoplasma contamination	Cells were negative for mycoplasma and routinely tested by Hoechst staining.
Commonly misidentified lines (See ICLAC register)	No commonly misidentified cell lines were used

Animals and other organisms

Policy information about studies involving animals; ARRIVE guidelines recommended for reporting animal research

Laboratory animals	Ten-week-old Slc:ddY male mice were obtained from Japan SLC, Inc.
Wild animals	*Provide details on animals observed in or captured in the field; report species, sex and age where possible. Describe how animals were caught and transported and what happened to captive animals after the study (if killed, explain why and describe method; if released, say where and when) OR state that the study did not involve wild animals.*
Field-collected samples	*For laboratory work with field-collected samples, describe all relevant parameters such as housing, maintenance, temperature, photoperiod and end-of-experiment protocol OR state that the study did not involve samples collected from the field.*
