# Supplementary material for: Nuclear lipid droplets derive from a lipoprotein precursor and regulate phosphatidylcholine synthesis
Source: Nat Commun. 2019 Jan 28;10:473. doi: 10.1038/s41467-019-08411-x (PMC6349838; doi:10.1038/s41467-019-08411-x)
Supplement: Supplementary file 10 — Description of Additional Supplementary Files [file 41467_2019_8411_MOESM10_ESM.doc]

**Supplementary Movie legends**

**Title: Supplementary Movie 1.
Description:** Huh7 cells treated with OA/TM for 48 h. The NR membrane (EGFP-LBR, green) and LDs (LipidTox Red, red) were observed at 2 min intervals. Disintegration of the NR membrane and relocation of an NR-lumenal LD to the nucleoplasm are observed (arrow). Selected frames are shown in Fig. 3c.

**Title: Supplementary Movie 2.
Description:** Huh7 cells treated with OA/TM for 48 h and then moved to a fresh OA/TM-free medium. The NR membrane (EGFP-LBR, green) and LDs (LipidTox Red, red) were observed at 2 min intervals. An NR-lumenal LD was observed to become nucleoplasmic as an LBR ring opens (arrow).

**Title: Supplementary Movie 3.
Description:** Huh7 cells were treated as in Supplementary Movie 2. The NR membrane (RFP-LBR, red) and perilipin-3-EGFP (green) were observed. Perilipin-3-EGFP was observed to occupy the entire surface of an NR-lumenal LD immediately after gap formation in the NR membrane (arrow).

**Title: Supplementary Movie 4.
Description:** Huh7 cells pretreated with OA for 24 h were observed continuously in the presence of OA for another 24 h at 2 min intervals. The NR membrane (EGFP-LBR); LDs (LipidTox Red). The NR membrane was observed in daughter cells immediately after mitosis. In contrast, nuclear LDs were regenerated only several hours after mitosis. Selected frames are shown in Fig. 5a and Supplementary Fig. 5a.

**Title: Supplementary Movie 5.** Huh7 cells pretreated with OA for 24 h were observed for **Description:** another 24 h in the presence of OA alone (left) or OA and MTPi (100 nM BAY 13-9952) (right) at 2 min intervals. Histone H2B-EGFP (green); LDs (LipidTox Red, red). Regeneration of nuclear LDs in daughter cells was observed several hours after mitosis in the absence of MTPi, but was scarcely observed in the presence of MTPi. Selected frames are shown in Fig. 5b.

**Title: Supplementary Movie 6.
Description:** Huh7 cells pretreated with OA for 24 h were observed for another 24 h in the presence of OA alone (left) or OA and MTPi (1 μM BAY 13-9952) (right). EGFP-NLSx3-HPos (red); histone H2B-mCherry (green). Nucleoplasmic LDs were regenerated in daughter cells several hours after mitosis, but this was suppressed significantly by MTPi. Selected frames are presented in Fig. 5c.
